# Analysis of interface mechanical properties between geotextiles and tailings during pull-out tests

**DOI:** 10.1371/journal.pone.0276543

**Published:** 2022-10-26

**Authors:** Changbo Du, Dongze Li, Fu Yi, Laigui Wang, Ben Niu

**Affiliations:** 1 College of Civil Engineering, Liaoning Technical University, Fuxin, China; 2 College of Architecture and Transportation, Liaoning Technical University, Fuxin, China; 3 School of Mechanics and Engineering, Liaoning Technical University, Fuxin, Liaoning, China; University of Vigo, SPAIN

## Abstract

Considering the strain-softening characteristics of the pull-out interface between geotextiles and tailings; to determine the interface interaction characteristics, this paper proposes a trilinear shear stress displacement softening model of geotextile-reinforced tailings. The obtained nonlinear governing equations were dimensionless, which were expressed in finite difference form. The results indicated that an accurate numerical solution could be obtained within a reasonable calculation time by discretizing the reinforcement length into 300 units. Three new dimensionless interaction terms, namely, the relative stiffness *α*, relative displacement *β*, and relative interface shear stiffness *η* of the reinforcement-tailings interaction, were introduced. In addition, an estimation method based on the approximate value of the relative stiffness *α* of the reinforcement-soil interface in the low-tensile-force displacement range was proposed. The interface shear stress range according to parameters *α*, *β*, and *η* was parameterized, and the normalization relationship between the tensile force and pull-out end displacement was determined. The numerical values calculated by the model were compared with the pull-out test results, demonstrating that the proposed model can accurately predict the pull-out behavior of the extensible reinforcement.

## 1 Introduction

In reinforcement engineering, the interface interaction characteristics of the reinforcement and soil play critical roles in the design and analysis of reinforced structures [1, 2]. Given that the stresses and displacements are gradually transferred from the pull-out end to the free end in pull-out tests, which can fully reflect the interaction mechanism of the reinforcement and soil, mechanical tests to evaluate the reinforced soil interface are generally adopted in pull-out tests [3].

Multiple scholars have derived a reinforcement–soil interface model based on pull-out tests [4–8]. Sobhi et al. [9] proposed an elastic–plastic shear stress–displacement pull-out interface model based on extensible reinforcement; Konami et al. [10] proposed a polymer strip elastic model based on the in-situ geosynthetic pull-out test; and Long et al. [11] used the parabola fitting curve to describe the non-uniform shear distribution of the reinforcement-soil interface. Gurung et al. [12] proposed a finite-difference-based expression to predict the pull-out resistance and reinforcement deformation in the pull-out test of geosynthetics and obtained prediction results that were consistent with the test results. Based on the linear elastic theory and under the same normal pressure, Yuan [13] proposed that the tensile force was a function of the reinforcement stiffness, the shear stiffness of the reinforcement–soil interface, the embedded length of reinforcement, and the pull-out displacement. Cui et al. [14] proposed a simplified theoretical model to investigate the hardening behavior of geobelts in pull-out tests; Xu et al. [15] conducted pull-out tests to analyze the interface properties of steel fibers embedded in an polyester and epoxy matrix, and the experimental results were fitted to an analytical model to obtain the interface shear strength. Huang et al. [16] theoretically derived a nonlinear interface model reflecting the effect of residual shear strength at the anchorage interface based on the double exponential curve interface model. Zhang et al. [17] conducted a series of pull-out tests to investigate the complex interaction mechanism between reinforcement nodes and the properties of the reinforcement interface and obtained a theoretical model for the ultimate pull-out resistance. Li et al. [18] used a hyperbolic model to simulate the front and back parts of the shear stress displacement curve. Wang et al. [3] studied the displacement and strain characteristics of geogrids with different embedded lengths and revealed the reinforcement–soil interaction mechanism. Although the abovementioned reinforcement–soil pull-out interface model can more accurately predict the pull-out mechanical properties of reinforcement, limited research has been conducted on the strain-softening mechanical model of the reinforcement–soil pull-out interface. Given that tailings are non-viscous artificial bulk materials, their mechanical behavior is significantly different from that of natural sand, which exhibits a relatively round particle shape.

Based on an indoor pull-out test of high-ductility geotextiles and tailings, the aim of this study was to investigate the strain softening and plastic flow phenomena at the pull-out interface of geotextile-reinforced tailings. A three-stage elastoplastic model of the shear stress–displacement relationship was used to predict the mechanical properties of the pull-out interface of geotextile-reinforced tailings. The variation laws of the nonlinear displacement and the tensile force of the reinforcement in the pull-out tests were analyzed, and the prediction results of the model were compared with the test results to verify the accuracy of the derived reinforcement–tailings pull-out interface model.

## 2 Materials and methods

### 2.1 Pull-out test apparatus

**Fig 1:**
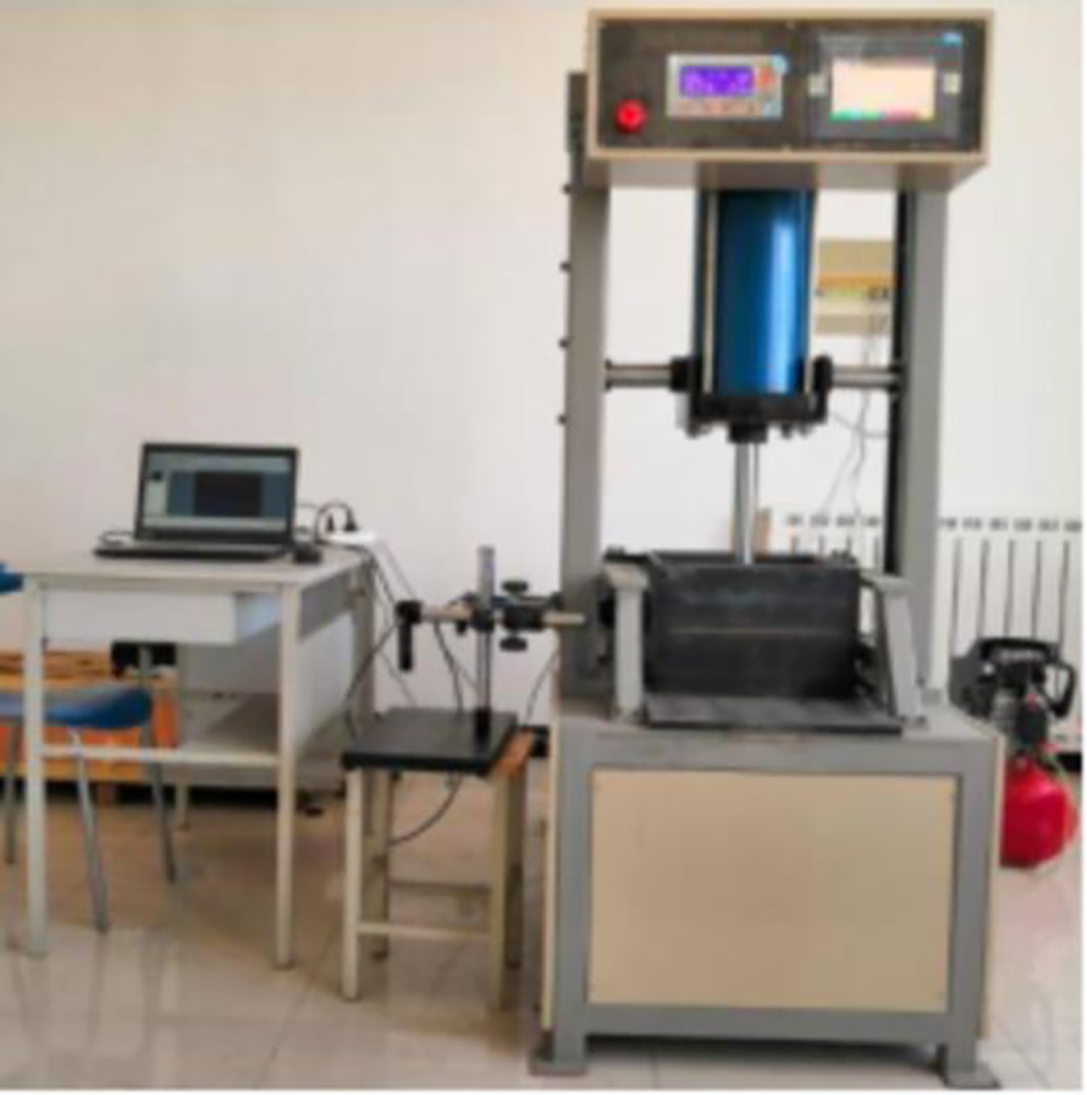
Pull-out test device.

The test apparatus was adapted from the YT1200 geosynthetic direct shear pull-out test system produced by the Nanjing Huade Instrument Company, which mainly consisted of a test chamber, vertical loading system, horizontal loading system, and data acquisition system, as shown in Fig 1. The inner diameter of the pull-out test chamber was set as 300 × 300 × 220 mm, with a narrow slit with dimensions of 300 × 10 mm at the front and rear of the test tank for the lead out of the geosynthetic. The vertical loading system consisted of a pneumatic loading system with a pressure transducer that applied the overlying pressure via a counterforce frame with a pressure-bearing plate with dimensions of 295 × 295 ×10 mm on top of the pneumatic loading system; such that the overlying pressure was uniformly applied. A horizontal loading system using rate-controlled pulling motors with tensile force sensors was used to apply a uniform speed to the test and measure the test force. This tester was equipped with a control panel (see Fig 1); which reflected the test results on the display in real time and realized real-time monitoring of the test data for analysis or the timeous termination of the test upon the occurrence of issues.

### 2.2 Tailings fill and geotextile parameter index

The tailings sand filler used in the test was obtained from the Fengshuigou tailings pond of the Qidashan concentrator of the Angang Mining Group, China. The permit for taking tailing sand samples was permitted by the Qidashan concentrator of the Angang Mining Group. Dry tailings sand was used to reduce the influence of moisture on the test results. The density of the tailings was 1.83 g/cm^3^, and the water content was 3.75%. The physical properties of the tailings were as follows: effective particle size *d*_10_ = 0.10 mm; median particle size *d*_30_ = 0.19 mm; and restricted particle size *d*_60_ = 0.30 mm. The particle size distribution of the tailings is shown in Table 1. The calculations showed that the tailings unevenness and curvature coefficients were *C*_u_ = 3.5 and *C*_c_ = 1.2, respectively. The *C*_c_ value is between 1 and 3, which indicates that the tailings were of poor gradation.

**Table 1:** Grain gradation of tailings.

Grain composition/%					Restricted particle size/mm			*C*_u_	*C*c
0.6–1.18 /mm	0.3–0.6 /mm	0.15–0.3 /mm	0.075–0.15 /mm	< 0.075 /mm	*d*_60_	*d*_30_	*d*_10_		
6.81	30.89	42.98	12.98	5.15	0.3	0.19	0.10	3.00	1.20

**Fig 2:**
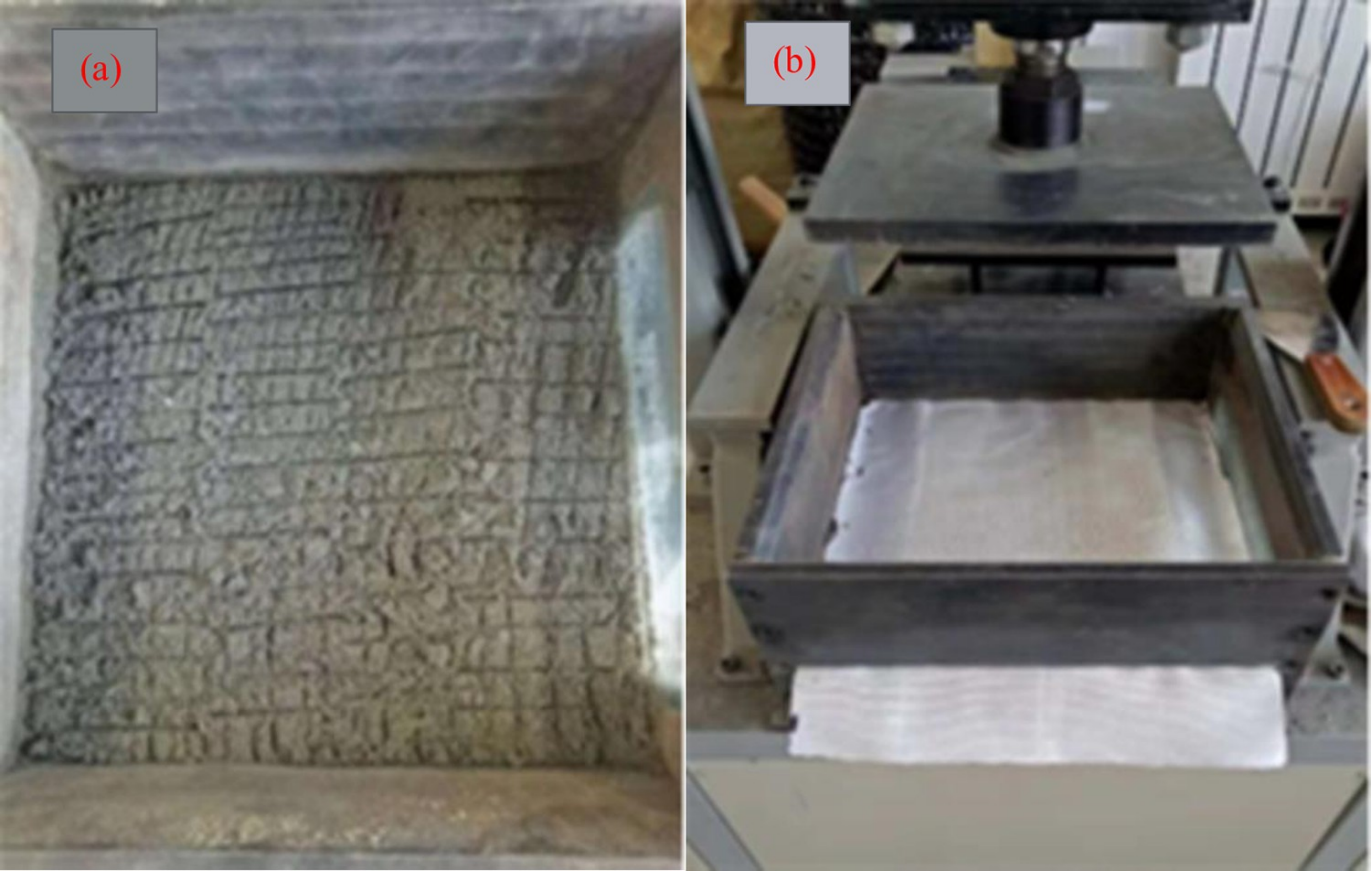
Pull-out test process: (a) Scraping treatment of the contact surface between geotextiles and tailing; (b) Geotextiles are spread through narrow slits and in the test chamber.

As shown in Fig 2, the geosynthetic material used in the test was a short-fiber needle-punched geotextile. In particular, this highly extensible geotextile is highly applicable to various reinforcement projects, and its specific performance parameters are shown in Table 2.

**Table 2:** Performance parameters of the geotextiles.

Mechanical parameters of the needled short-fiber geotextiles	Performance index
Longitudinal and transverse breaking strength/(kN/m)	30
Elongation corresponding to longitudinal and transverse standard strength /%	40–80
CBR bursting strength/kN	≥ 13
Longitudinal and transverse tearing strength/kN	≥ 12
Equivalent aperture/mm	0.05~0.2
Vertical permeability coefficient/(cm/s)	1.0×10^~3^
Thickness/mm	4.2

### 2.3 Test method and procedure

Pull-out tests of the geotextiles were carried out under four different overlay pressures, and 1–3 parallel tests were carried out for each set of tests to reduce the dispersion of the test results. To reduce the boundary effects, the two sides of the pull-out test chamber were evenly coated with lubricating oil. The shear rate of the pull-out test was set at 2 mm/min to eliminate the influence of the test rate on the results. The test was performed with strict reference to the geosynthetic testing procedure (SL-2012) [19]. During the tests, the relative density of the tailings sand was used to control the amount of sand filling in the test tank. The samples were compacted in layers (four layers in total) to ensure that the relative compactness of each group of tests was approximately 90%. The specific pull-out test procedure was as follows:

1. Cut out the geotextiles sample for the test, clean the test chamber, and apply engine oil on the side wall of the test chamber for lubrication for preparation for the test.
2. Calculate the amount of tailings filled in the first layer, put it into the drawing box, and compact it with the vertical loading system; then measure the height after compaction to match the calculated value. Subsequently, shave its surface and continue to pack the second layer of tailings and compact it to the calculated value. Make sure that the relative density of all loaded tailings in the test is 90%. The tailings surface is scraped (Fig 2A) to ensure a close contact at the contact interface. The geotextiles are penetrated through narrow slits on the front and rear side walls of the test chamber (Fig 2B), and its edges are strictly controlled so that they are aligned with and parallel to the test chamber and are firmly connected to the fixture.
3. The third and fourth layers of tailings are filled in the upper half of the test chamber in the same way as the lower half of the test chamber. The level of the upper surface of the fill is strictly controlled to ensure that the vertical pressure is evenly applied; then, apply a predetermined vertical pressure and start the test after 20 min of consolidation.
4. Start the pre pull-out and stop the pull-out when the pull-out force is 0.15 kN, so as to tighten the geotextiles. Then, the instrument is zeroed and the pull-out test is started according to the pull-out speed (2 mm/min). During the test, the pulling force and pulling displacement are recorded.
5. Stop the test after the peak pulling force is reached or when the pulling displacement is greater than 40 mm.

### 2.4 Analysis of the pull-out test results

**Fig 3:**
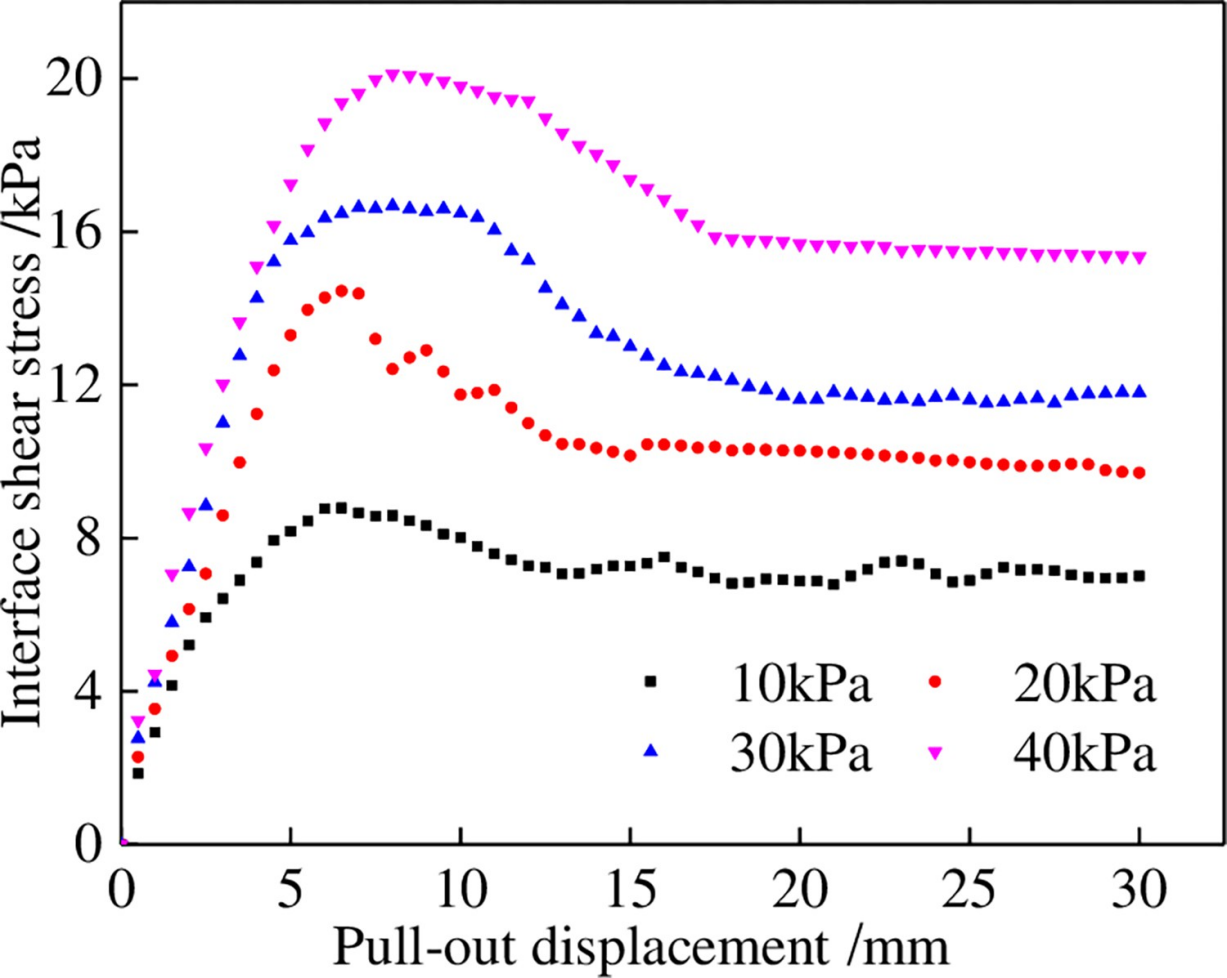
Pull-out test results between geotextiles and tailings.

**Fig 4:**
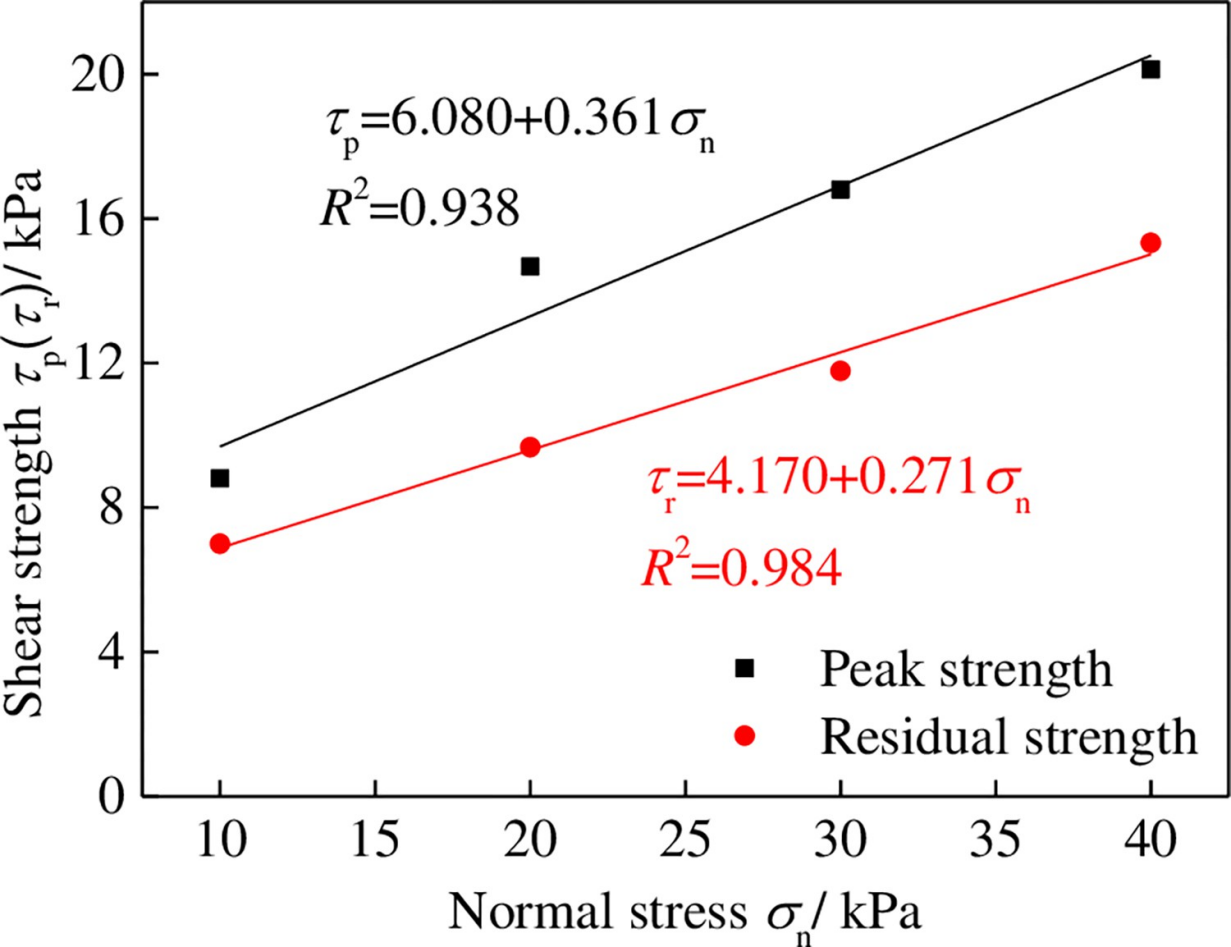
Relationship between friction strength and normal stress.

The pull-out test results for the geotextiles and tailings under different normal stresses are shown in Fig 3. The interface shear stress increased rapidly with the pull-out displacement and decreased after reaching the peaks. Subsequently, the interface shear stress remained stable with an increase in the pull-out displacement. The pull-out test curves for these highly extensible geotextiles are roughly characterized by general strain softening. The relationship between the shear strength and the normal stress of the geotextiles under the pull-out test is shown in Fig 4. The shear strength and normal stress of the geotextiles in the peak and residual states are linearly correlated, with correlation coefficients above 93%. The pseudo-cohesion and pseudo-friction angle of the interface parameters in the peak and residual states of geotextiles are 10.22 kPa and 21.31° and 6.08 kPa and 19.85°, respectively.

## 3 Results and discussion

### 3.1 Shear stress–displacement relationship model

**Fig 5:**
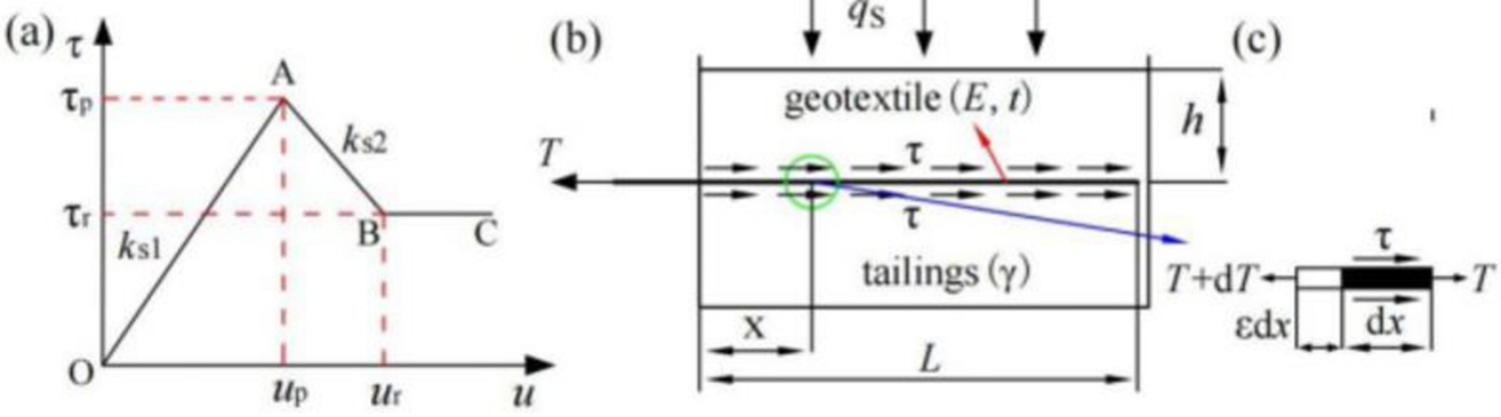
Schematic of the pull-out interface between geotextiles and tailings: (a)Trilinear relationship between the interface shear stress and displacement; (b) overall reinforcement stress; (c) force analysis of the reinforcement micro-element.

According to the test results, the shear stress–displacement (*τ*−*u*) test curve at the reinforcement–tailings interface can be approximated as a straight line until the peak strength was reached, after which plastic softening and plastic flow were exhibited. In this study, this curve feature was simplified to a three-stage linear shear stress–displacement relationship (*τ* for shear stress, and *u* for displacement), as shown in Fig 5(A). In the first stage, the OA section represents the *τ*−*u* relationship before the shear stress reaches its peak in a straight line. In the second stage, the AB section represents the strain softening of the geotextiles in a straight line. In the third stage, the BC section represents the plastic flow of the geotextiles in a horizontal straight line. In Fig 5(A), the *k*_s1_ is the slope of the OA section (interface shear stiffness), *τ*_p_ is the peak shear stress reached by the reinforcement, *u*_p_ is the corresponding displacement (*τ*_p_ = *k*_s1_*u*_p_), *k*_s2_ is the slope of the AB section (interface shear stiffness), *τ*_r_ is the peak shear stress reached by the reinforcement, and *u*_r_ is the corresponding displacement. The ratio between *τ*_r_ and *τ*_p_ is defined as the damage ratio *R*_f_ [8], i.e., *R*_f_ = *τ*_r_/*τ*_p_, which are generally between 0.5 and 1.0.


$$\tau =\{\begin{array}{l} k_{s1}u,\;0\leq u\leq u_{p}\\ k_{s2}(u-u_{p})+k_{s1}u_{p},\;u_{p}<u\leq u_{r}\\ R_{f}k_{s1}u_{p},\;u>u_{r} \end{array}$$ (1)

### 3.2 Interface stress analysis

The pull-out test diagram between geotextiles and tailings is shown in Fig 5(B) The bottom and lateral boundaries of the test tank were fixed during the test, and a vertical overburden pressure was applied to the pressure-bearing plate. The length and thickness of the reinforcement are *L* and *t*, respectively, and the tensile modulus is *E*. The shear stress of the reinforcement at *x* is *τ*, and a microcellular body of length d*x* was considered for analysis, as shown in Fig 5(C). The width is the unit width of the reinforcement, which does not consider the boundary effect of the reinforcement. The following can be obtained based on the stress equilibrium:

dT−2τ(dx+εdx)=0 (2)

where *T* is the unit-width tensile force of the reinforcement at *x*, *εdx* is the unit deformation length of the microelement, and *ε* is the strain.

According to the definition of strain, the strain in a geotextile at *x* can be expressed as follows:

$$\varepsilon =-\frac{du}{dx}$$ (3)

where *u* is the relative displacement of the reinforcement soil at *x*.

The strain is assumed to be linearly related to the tensile force per unit width, as follows:

$$\varepsilon =\frac{T}{Et}$$ (4)

From Eqs (2)–(4), the following is obtained:

$$Et\frac{d^{2}u}{dx^{2}}+2\tau (\frac{du}{dx}-1)=0$$ (5)

Substituting Eq (1) into Eq (5) and making it dimensionless, and let *U* = *u*/*u*_p_ and *X* = *x*/*L*, we can obtained:

$$\left\{ \begin{array}{l} \frac{d^{2}U}{dX^{2}}+\alpha U(\beta \frac{dU}{dX}-1)=0,\;0\leq U\leq 1\\ \frac{d^{2}U}{dX^{2}}+\alpha [1+\eta (U-1)](\beta \frac{dU}{dX}-1)=0,\;1<U\leq [(R_{f}-1)/\eta +1]\\ \frac{d^{2}U}{dX^{2}}+\alpha R_{f}(\beta \frac{dU}{dX}-1)=0,\;U>[(R_{f}-1)/\eta +1] \end{array}\right.$$ (6)

where $\alpha =\frac{2k_{s1}L^{2}}{Et}$ is the relative stiffness; $\beta =\frac{u_{p}}{L}=\frac{\tau_{p}}{k_{s1}L}$ is the relative displacement; and $\eta =\frac{k_{s2}}{k_{s1}}$ is the relative interface shear stiffness. In general, |*k*_*s*1_|>|*k*_*s*2_| [20], so the value range of the relative interface shear stiffness is −1<*η*<0.

From the boundary conditions of the reinforcement under stress, the following is obtained:

$$\left\{ \begin{array}{l} x=0,\;\varepsilon =-\frac{T_{0}}{Et}\Rightarrow \frac{\mathrm{dU}}{dX}=-\alpha T^{*},X=0\\ x=L,\;\varepsilon =0,\;\Rightarrow \frac{\mathrm{dU}}{dX}=0,\;X=1 \end{array}\right.$$ (7)

where T* = *T*_0_/*T*_m_ denotes the dimensionless tensile force, *T*_0_ denotes the tensile force at *X* = 0, and *T*_m_ = 2*τ*_p_*L* denotes the maximum tensile force.

### 3.3 Numerical solutions

**Fig 6:**
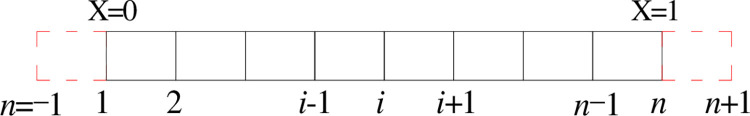
Schematic of the simulation reinforcement discretization.

Eq (6) is a second order nonlinear differential equation that cannot be solved using analytical methods. By discretizing the reinforcement into *n* units (each of length Δ*L* = *L*/*n* or Δ*X* = 1/*n*), as shown in Fig 6, and then expressing the derivatives in finite difference form; Eq (6) can be re-written as an equation with *i* nodes as follows:

$$\left\{ \begin{array}{l} \frac{U_{i+1}-2U_{i}+U_{i-1}}{{\left(\Delta X\right)}^{2}}+\alpha U_{i}\left[\beta (\frac{U_{i+1}-U_{i-1}}{2\Delta X})-1\right]=0,\;0\leq U_{i}\leq 1\\ \frac{U_{i+1}-2U_{i}+U_{i-1}}{{\left(\Delta X\right)}^{2}}+\alpha \left[1+\eta (U_{i}-1)\right]\left[\beta (\frac{U_{i+1}-U_{i-1}}{2\Delta X})-1\right]=0,\;1<U\leq \left[(R_{f}-1)/\eta +1\right]\\ \frac{U_{i+1}-2U_{i}+U_{i-1}}{{\left(\Delta X\right)}^{2}}+\alpha R_{f}\left[\beta (\frac{U_{i+1}-U_{i-1}}{2\Delta X})-1\right]=0,\;U>[(R_{f}-1)/\eta +1] \end{array}\right.$$ (8)

The normalized displacement of Node *i* can be obtained by the following iterations:

$$U_{i}=\{\begin{array}{l} \frac{U_{i+1}+U_{i-1}}{2-\alpha C_{1}/n^{2}},\;0\leq U_{i}\leq 1\\ \frac{U_{i+1}+U_{i-1}+\alpha (1-\eta )C_{1}/n^{2}}{2-\alpha \eta C_{1}/n^{2}},\;1<U\leq [(R_{f}-1)/\eta +1]\\ \frac{U_{i+1}+U_{i-1}+\alpha R_{f}C_{1}/n^{2}}{2},\;U>[(R_{f}-1)/\eta +1] \end{array}$$ (9)

where $C_{1}=\beta n\frac{U_{i+1}-U_{i-1}}{2}-1$

To determine the displacements at Nodes *i* = 1 and *i* = *n* + 1, two virtual nodes *i* = 0 to the left of *i* = 1 and *i* = *n* + 1 to the right of Node *i* = *n* are assumed, and the displacements at these nodes can be derived from the boundary condition (Eq 7):

$$\left\{ \begin{array}{l} U_{-1}=U_{1}+(2\alpha T^{*})/n\\ U_{n-1}=U_{n+1} \end{array}\right.$$ (10)

With *U*_0_ and *U*_*n*+2_, the normalized displacements at Nodes *i* = 1 and *i* = *n* + 1 can be obtained from Eq (9). The strain and normalized tensile force of the reinforcement at Node *i* can then be obtained from the known displacements of the reinforcement along its length, as follows:

$$\varepsilon_{i}=n\frac{U_{i-1}-U_{i+1}}{2}$$ (11)

$$T_{i}^{*}=n\frac{U_{i-1}-U_{i+1}}{2\alpha }$$ (12)

### 3.4 Estimation of model parameter *α*

The first approximation of parameter *α* can be estimated by ignoring $\beta \frac{dU}{dX}\to 0$ in Eq (6) at a lower tensile force. However, the value at a higher tensile force may vary slightly according to slope *k*_s1_ in the *τ*−*u* curve response. For the initial estimation, the first equation in the set of Eq (6) can be approximated as follows:

$$\frac{d^{2}U}{dX^{2}}-\alpha U=0,\;0\leq U\leq 1$$ (13)

The closed solution of Eq (13) is expressed as follows:

$$U=C_{1}\exp(-\sqrt{\alpha }X)+C_{2}\exp(\sqrt{\alpha }X),\;0\leq U\leq 1$$ (14)

The differential equation is obtained from Eq (14) as follows:

$$\frac{dU}{dX}=-\sqrt{\alpha }[C_{1}\exp(-\sqrt{\alpha }X)-C_{2}\exp(\sqrt{\alpha }X)]$$ (15)

According to the boundary condition (Eq (7), the following is obtained:

$$\left\{ \begin{array}{l} X=0\;\frac{\mathrm{dU}}{dX}=-\alpha T^{*}\\ X=1\;\frac{\mathrm{dU}}{dX}=0 \end{array}\Rightarrow \{\begin{array}{l} C_{1}=\frac{\exp(\sqrt{\alpha })}{\exp(\sqrt{\alpha })-\exp(-\sqrt{\alpha })}\sqrt{\alpha }T^{*}\\ C_{2}=\frac{\exp(-\sqrt{\alpha })}{\exp(\sqrt{\alpha })-\exp(-\sqrt{\alpha })}\sqrt{\alpha }T^{*} \end{array}\right.$$ (16)

Substituting Eq (16) into Eq (14) yields the following:

$$U=\frac{\cosh\sqrt{\alpha }(1-X)}{\sinh(\sqrt{\alpha })}\sqrt{\alpha }T^{*}\;0\leq U\leq 1$$ (17)

The reinforcement material was at the pull-out end (at *X* = 0), which is expressed by Eq (17) as follows:

$$U=\frac{u}{u_{p}}=(\frac{\sqrt{\alpha }T_{0}}{T_{m}})(\coth\sqrt{\alpha })\;0\leq U\leq 1$$ (18)

Namely,

$$k_{0}=\frac{u}{T_{0}}=(\frac{u_{p}}{2\tau_{p}L})[\frac{\sqrt{\alpha }}{\tanh\sqrt{\alpha }}]=\frac{L}{\sqrt{\alpha }Et\tanh\sqrt{\alpha }}$$ (19)

Eq (19) expresses the initial slope (*k*_0_ = *u*/*T*_0_) of the displacement with respect to the tensile force. The initial slope *k*_0_ is determined by the physical properties of the reinforcement; namely, *E*, *t*, *L*, and the interface shear stiffness *k*_s1_. Moreover, *E*, *t*, and *L* are known; thus, the initial slope *k*_0_ of the tensile force–displacement test curve can be estimated from *k*_s1_. Eq (19) is dimensionless and can be used to obtain the following:

$$k_{0}\frac{Et}{L}=\frac{1}{\sqrt{\alpha }\tanh\sqrt{\alpha }}=f(\alpha )$$ (20)

**Fig 7:**
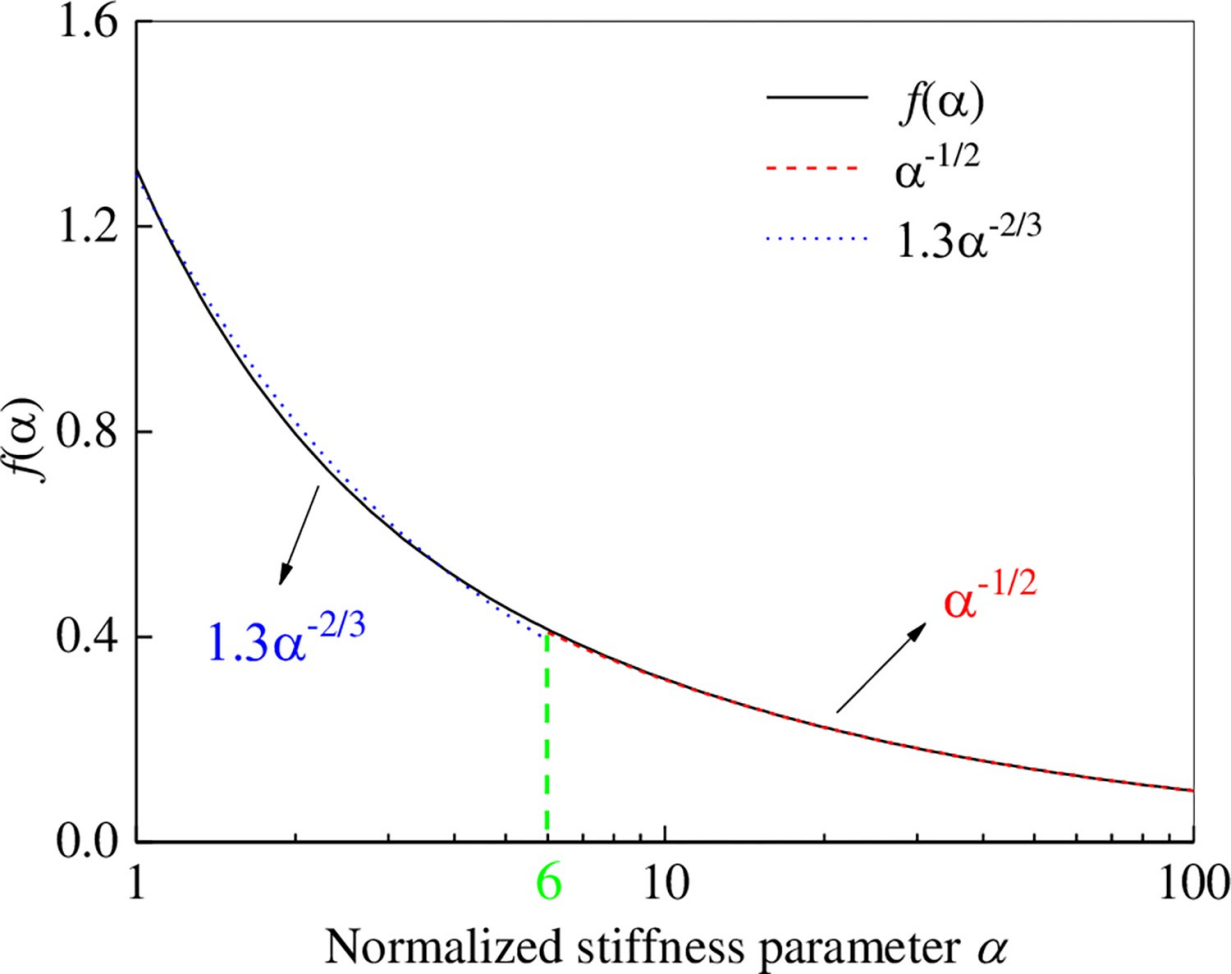
Relationship between *f*(*α*) and *α*.

Eq (20) presents an estimate of the value of *α* based on the tensile force-displacement test results. The values of *α* and *k*_s1_ under different test conditions can be estimated by drawing the Origin image of function *f*(*α*). The relationship between *f*(*α*) and *α* in Eq (20) is shown in Fig 7. When *α* ≤ 6, *f*(*α*) is approximately equal to 1.3*α*^−2/3^; when *α* > 6, *f*(*α*) is almost equal to*α*^−1/2^.

### 3.5 Analysis of the impact of model parameters

Using numerical solutions (8)–(12), the solutions for the normalized displacement, strain, and normalized tensile force along the length of the reinforcement material can be obtained, and the accuracy of the solutions can be verified by varying the number of units into which the reinforcement is discretized *n*. All further calculations were carried out using *n* = 300. The model parameters accept the following range of values: T* = 0−1.0, *R*_f_ = 0.5−1, *η* = −1.0−0, *α* = 2−200, and *β* = 0.001−0.1 (Table 3). The product $\alpha \beta =(T_{m}/Et)=2(c_{p}+\sigma_{n}\tan\phi_{p})L/Et$ of parameters *α* and *β* is only a function of the maximum tensile force and stiffness of the reinforcement material and is independent of the interface shear stiffness *k*_s1_.

**Table 3:** Range of model parameters.

Parameter	*R*_f_	*η*	T*	*α*	*β*
Selection range	0.5–1	-1–0	0–1	2–200	0.001–0.1
Base value	0.7	-0.4	1	20	0.01

**Fig 8:**
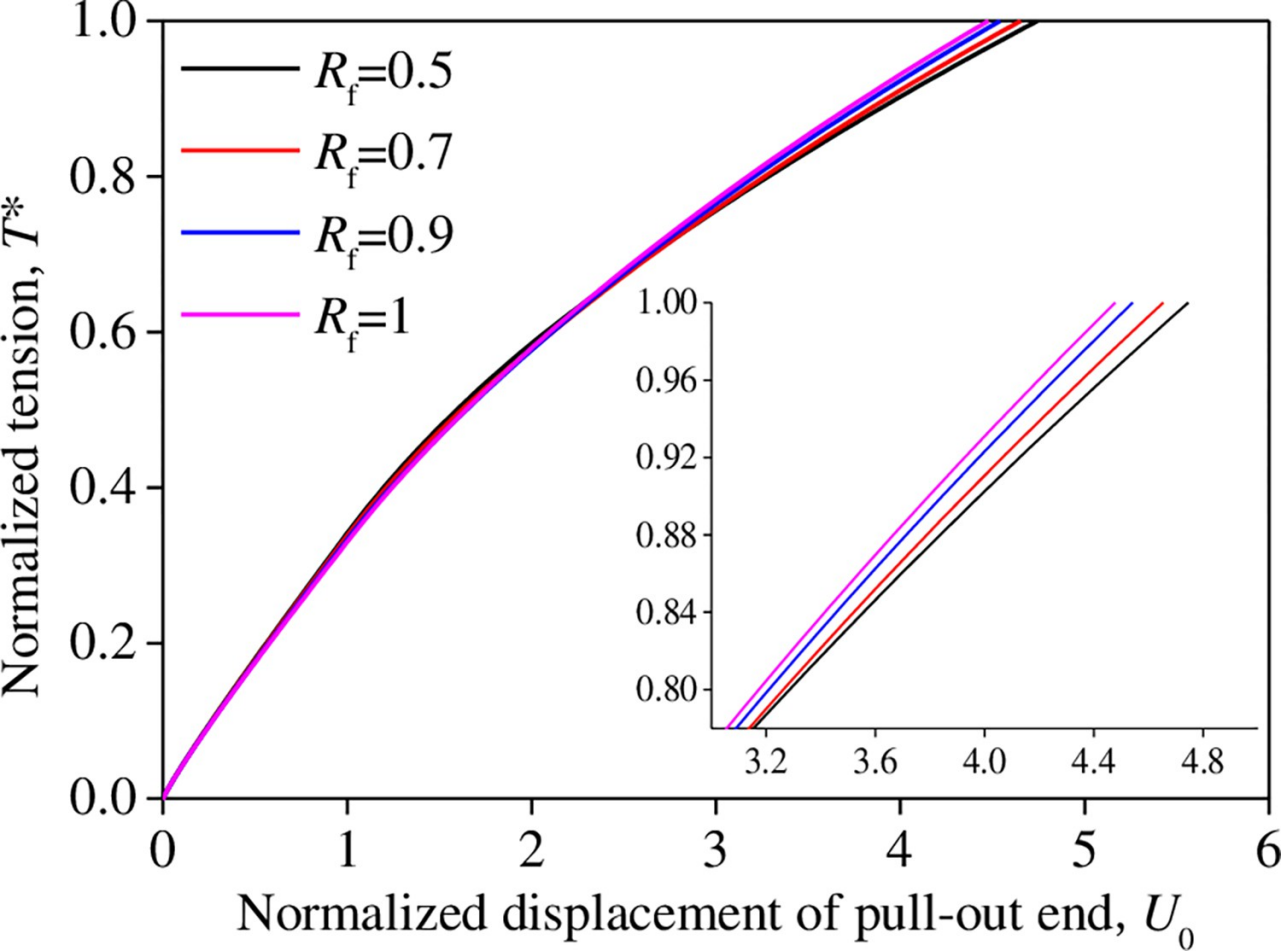
Influence of *R*_f_ on the T*–*U*_0_ relationship.

**Fig 9:**
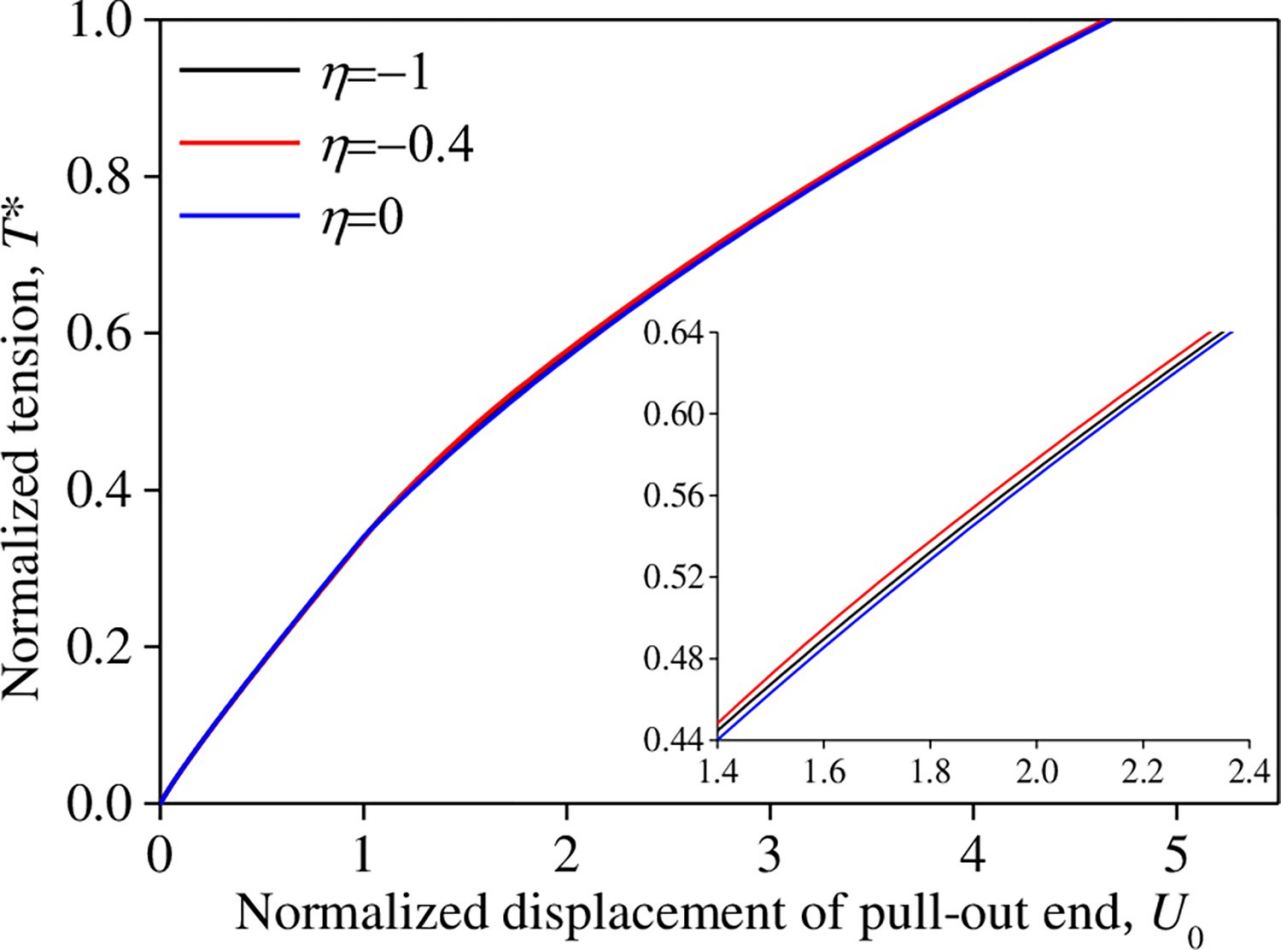
Influence of *η* on the T*–*U*_0_ relationship.

For different damage ratios *R*_f_ and relative interface shear stiffness *η* condition*s*, the variation of the normalized displacement *U*_0_ at the pull-out end (*X* = 0) with the normalized tensile force T* is shown in Figs 8 and 9. *R*_f_ and *η* exhibited an insignificant influence on the *U*_*0*_–T* curve. This indicates that the plastic characteristic parameters of the interface test curve between the reinforcement and tailings after reaching the peak strength have a slight influence on the shear stress–displacement relationship model.

**Fig 10:**
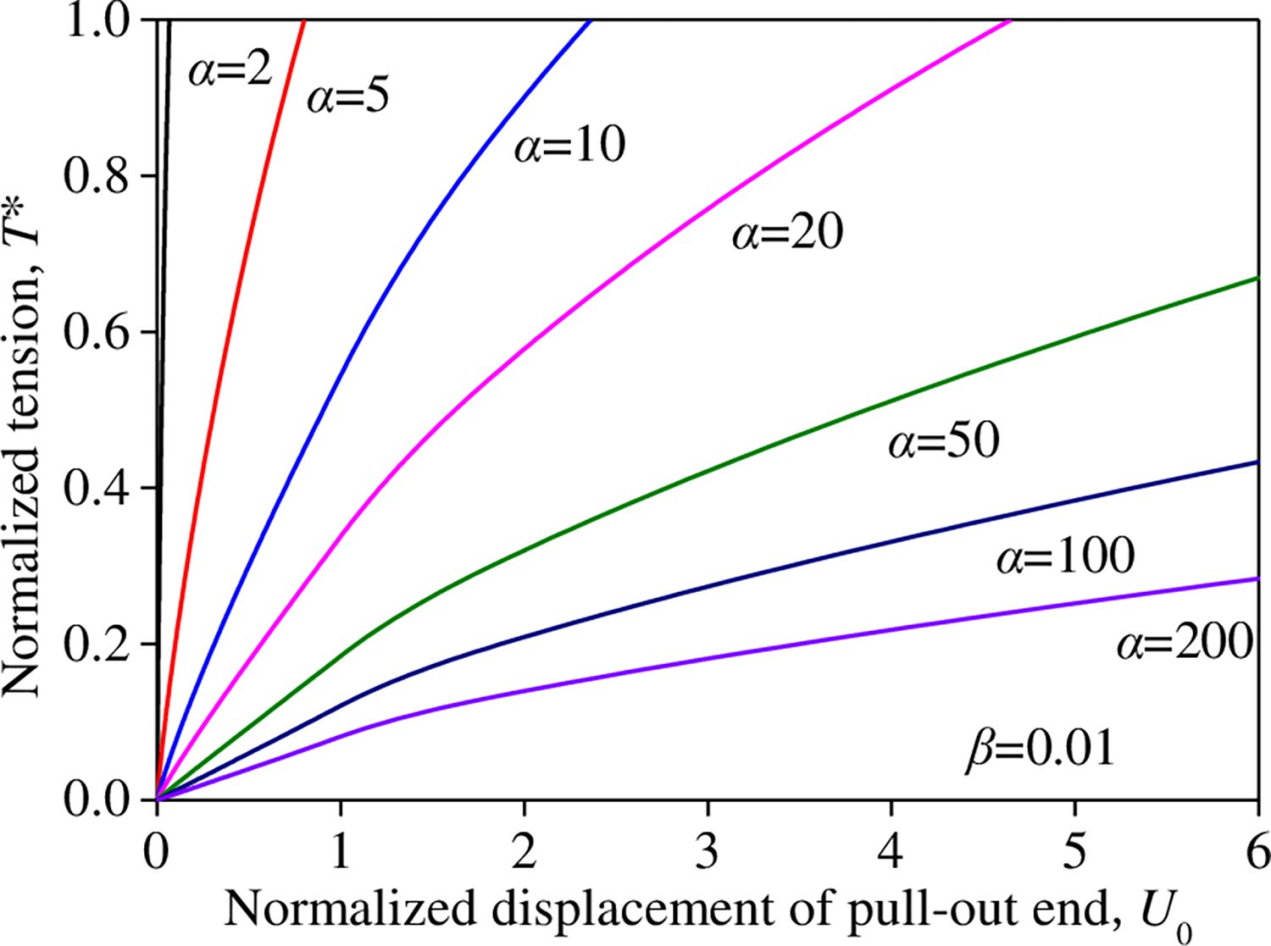
Influence rule of *α* on the T*–U_0_ relation (*β =* 0.01).

When *β* = 0.01, the normalized displacement *U*_0_ at the pull-out end (*X* = 0) varied with the normalized tensile force T* for different relative stiffnesses *α*, as shown in Fig 10. At constant *β* = 0.01, the displacement *U*_0_ increased significantly with an increase in *α* for any given normalized tensile force T*, and the tensile force T* decreased significantly with an increase in *α* for any given normalized displacement *U*_0_. When T* < 0.5, the normalized displacement *U*_0_ increased linearly with the normalized tensile force T*. When T*→1.0 for T* = 0.6; *α* = 2, 5, 10, 20, 50, 100, and 20 resulted in normalized displacements *U*_0_ of 0.946, 1.427, 2.249, 3.830, 7.995, 13.390, and 20.750, respectively.

**Fig 11:**
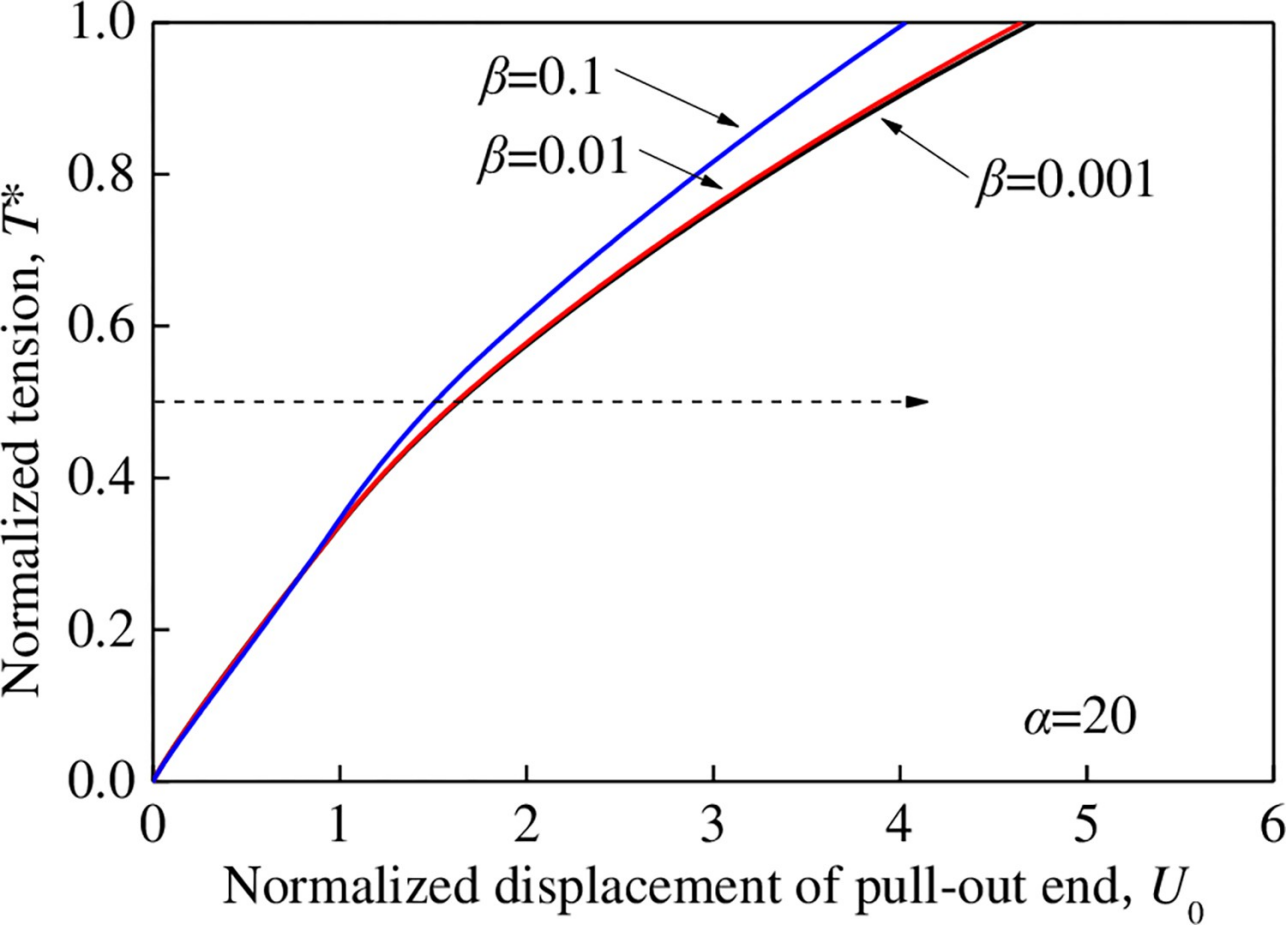
Influence rule of *β* on the T*–U_0_ relation (*α =* 20).

The variations in the normalized displacement *U*_0_ at the pull-out end (*X* = 0) with the normalized tensile force T* for different relative displacements *β* when *α* = 20 are shown in Fig 11. Changing the value of *β* (0.1, 0.01, and 0.001) while keeping *α* = 20 constant resulted in changes in the peak shear stress *τ*_p_. However, the interface shear stiffness *k*_s1_ was constant. The peak shear stress *τ*_p_ of the reinforcement increased with an increase in *β*. When T* = 0.5, *β* = 0.1, 0.01, and 0.001 yielded normalized displacements of 1.50, 1.63, and 1.65, respectively, and the normalized displacement increased with the normalized tensile force T*. When *β* < 0.01, the change in *β* minimally influenced the change of the T*–*U*_0_ curve.

**Fig 12:**
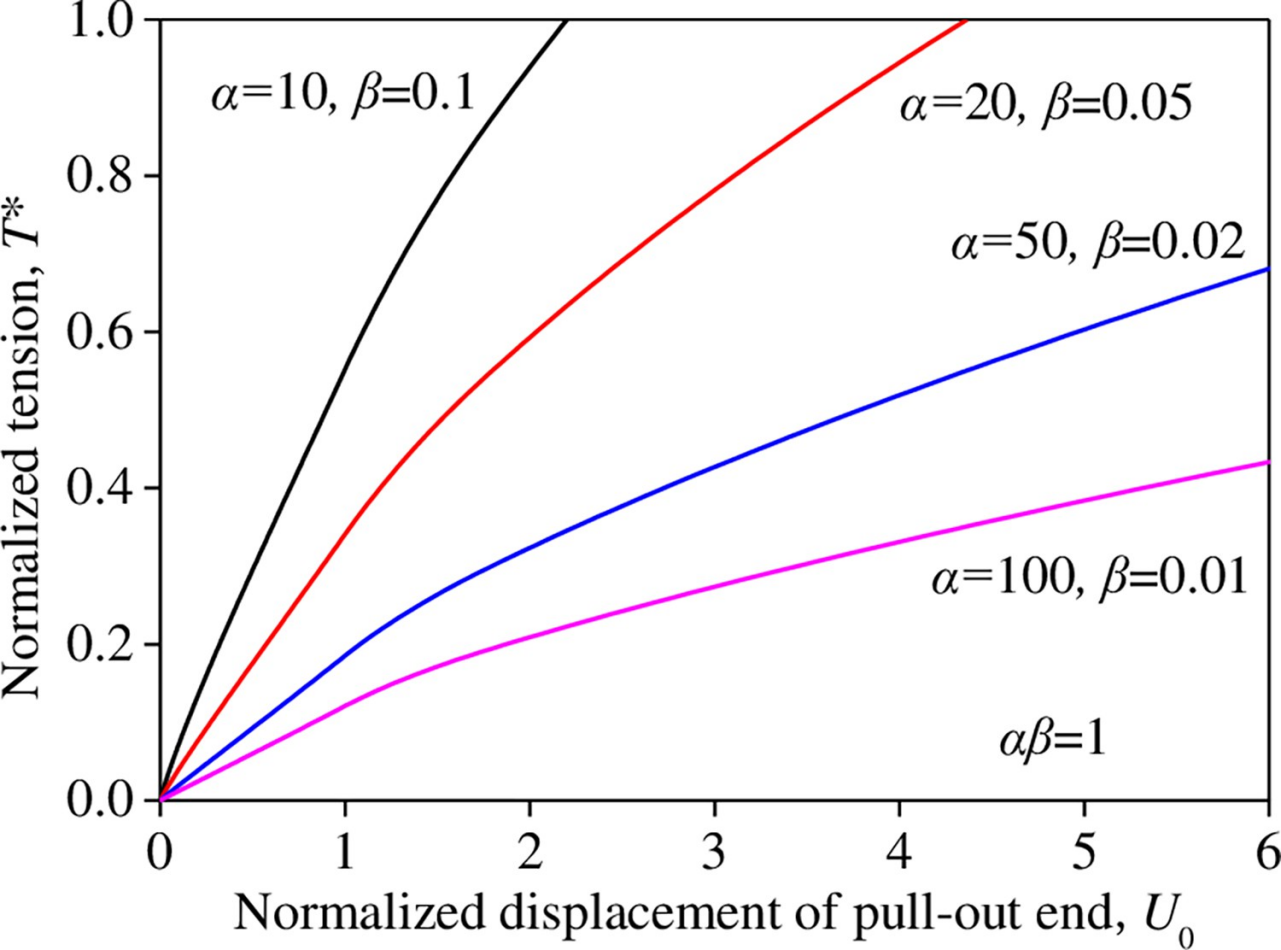
Influence of *αβ* on the T*–U_0_ relation (*αβ =* 1).

The variations of the normalized displacement *U*_0_ at the pull-out end (*X* = 0) and the normalized tensile force T* under a constant *αβ* = 1 are shown in Fig 12. For a constant *αβ* = 1 (*α* = 10, 20, 50, and 100; and *β* = 0.1, 0.05, 0.02, and 0.01), as the values of *α* increased and the values of *β* decreased, the non-linearity of the T*–U_0_ curve was more significant. The displacement *U*_0_ increased significantly for any given normalized tensile force T*, and the tensile force T* decreased significantly for any given normalized displacement *U*_0_.

**Fig 13:**
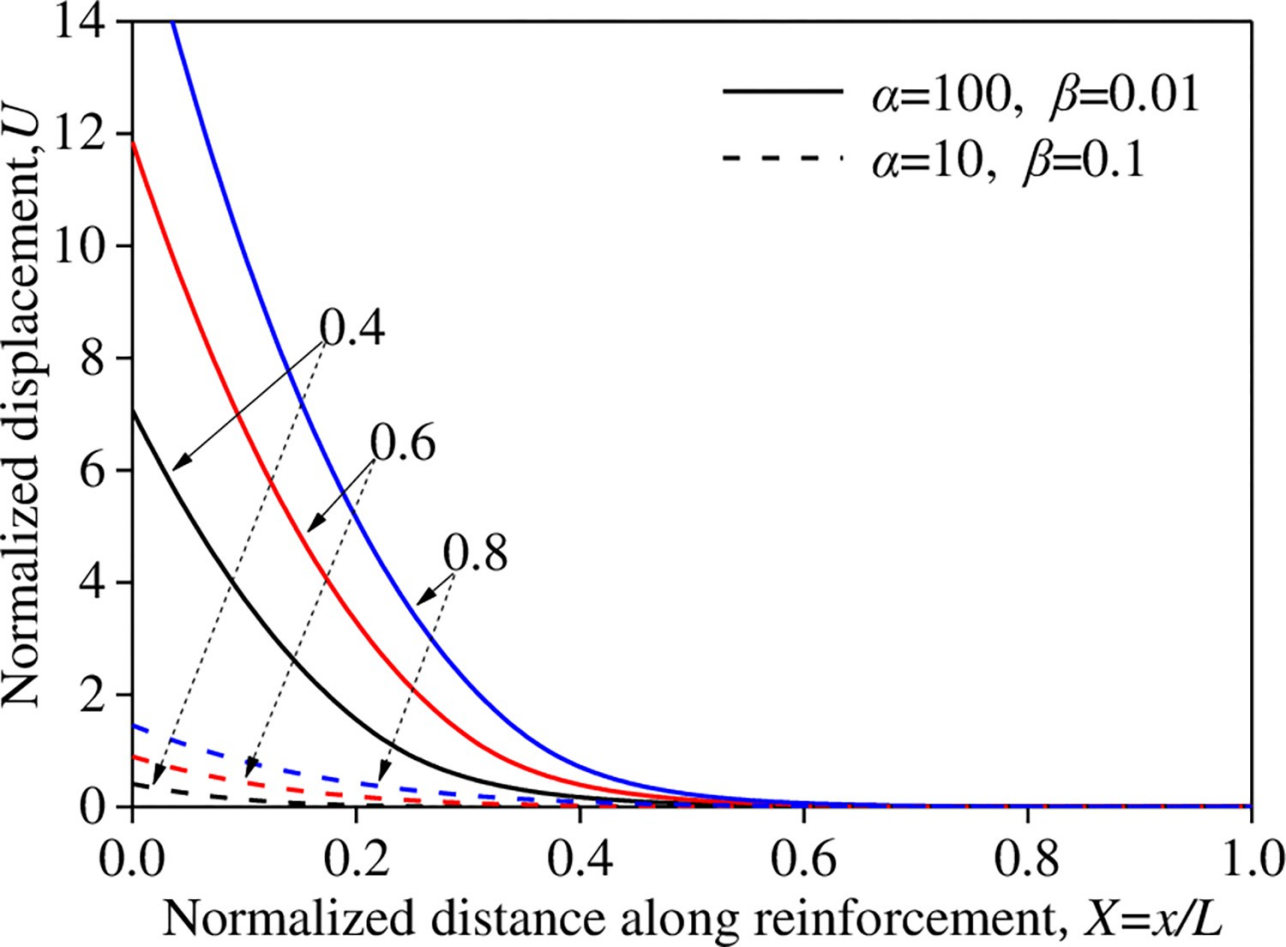
Influence of T* on *U–X* (*αβ =* 1).

Under a constant *αβ* = 1 (α = 100 and *β* = 0.01; and *α* = 10 and *β* = 0.1), the normalized displacement *U* varied with the normalized distance *X* along the reinforcement material for different normalized tensile forces T*, as shown in Fig 13. For higher *α* values and lower *β* values, the normalized displacement *U* decreased rapidly with an increase in the normalized distance *X* along the reinforcement material, indicating that only a portion of the reinforcement material effectively mobilized the pull-out resistance. Conversely, for smaller values of *α* and larger values of *β*, the full length of the reinforcement material mobilized the pull-out resistance. These results indicate that is necessary to consider the interface shear-stiffness stiffness *k*_s1_.

### 3.6 Model validation

**Fig 14:**
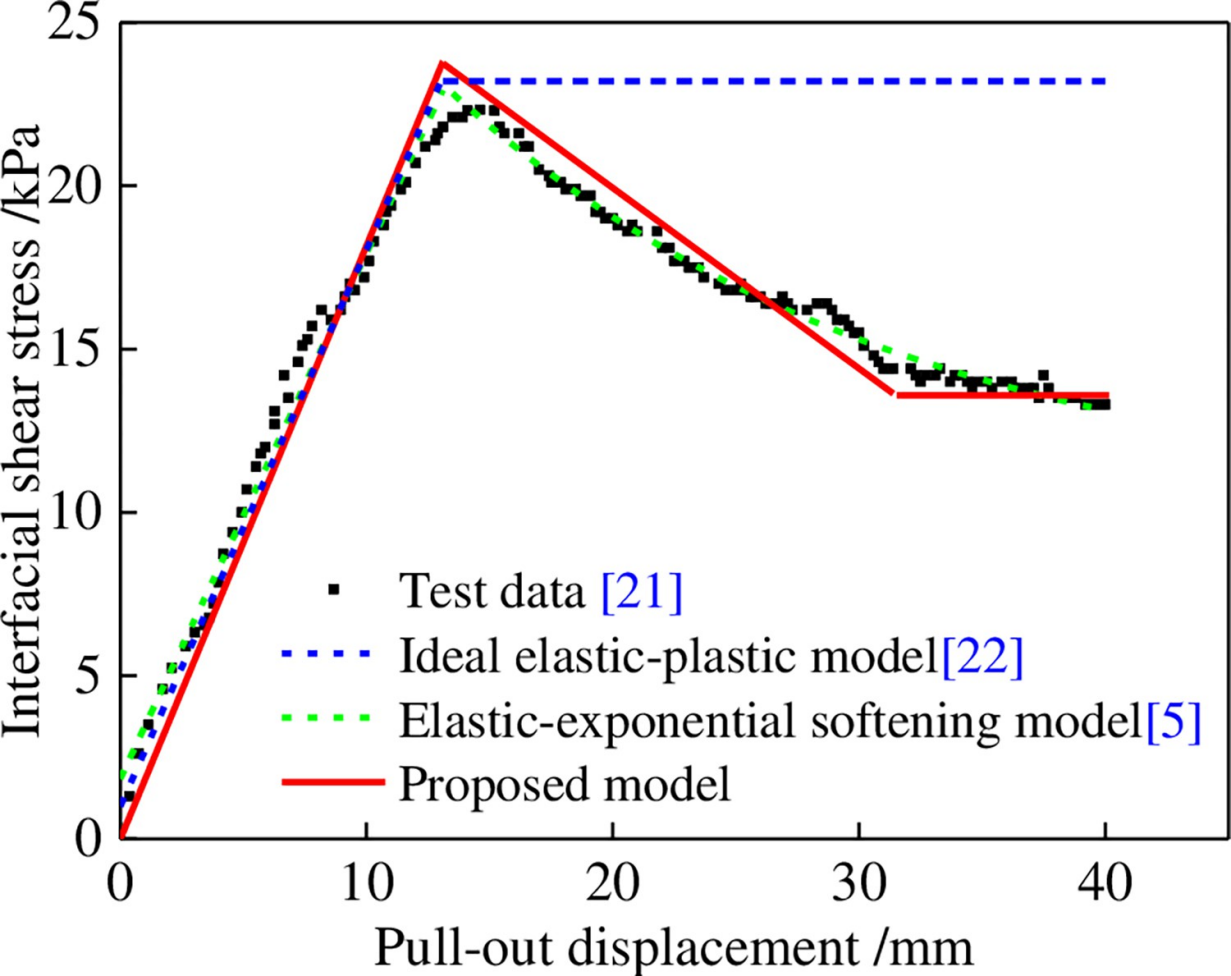
Comparative analysis of the model presented in this paper and two classical reinforced-soil interface models.

**Fig 15:**
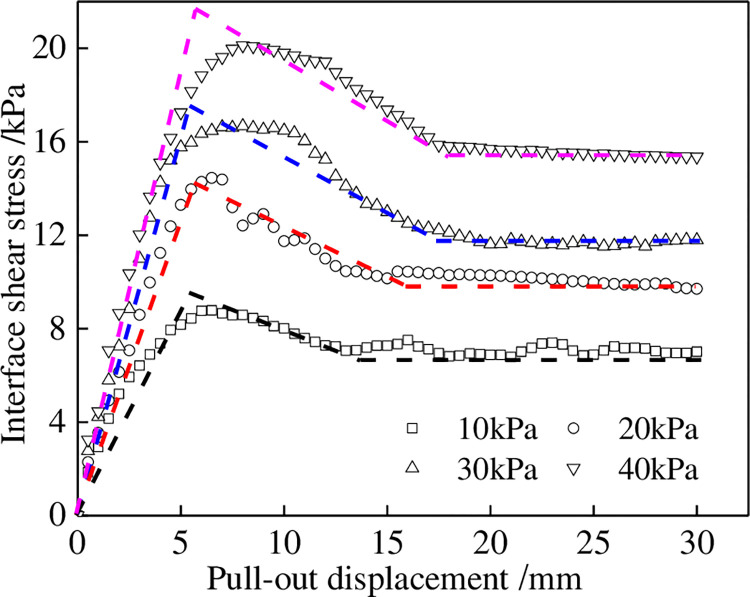
Comparison between the pull-out test results and model predicted results for geotextiles.

To validate the proposed model, the author [20] selected the pull-out test results of a one-way geogrid and cohesive soil under a normal stress of 100 kPa and compaction degree of 0.85 in Reference [21] to perform a simulation. Two classical reinforced-soil interface models, namely, the ideal elastic-plastic [22] and elastic-exponential softening [5] models, were used for comparison (see Fig 14). Based on the comparative results, it can be seen that the ideal elastic-plastic model cannot reflect the softening process of the interface, while the elastic-exponential softening model and the proposed trilinear shear stress-displacement softening model can better reflect the softening characteristics of the interface. However, the proposed trilinear softening model is simpler, more concise, and has better applicability. The pull-out test results for the geotextiles and tailings were simulated, and the simulation parameters are listed in Table 4. The simulation results are shown in Fig 15. The test data and simulation results were in good agreement, indicating that the model can appropriately simulate the pull-out characteristics of the interface of the softened reinforcement material.

**Table 4:** Simulated parameters of the pull-out tests.

*σ*_n_/kPa	*k*_s1_ /(MPa/m)	*k*_s2_ /(MPa/m)	*u*_p_*/*mm	*L*/m	*t*/mm	*E* /Mpa	*α*	*β*	*η*
10	1.76	-0.36	5.43	0.3	3.5	10	9.0514	0.0181	-0.2045
20	2.50	-0.43	5.70				12.8571	0.0190	-0.1720
30	3.21	-0.48	5.45				16.5086	0.0182	-0.1495
40	3.78	-0.51	7.75				19.4400	0.0258	-0.1349

## 4 Conclusions

1. A trilinear shear stress displacement softening model of geotextile-reinforced tailings was developed in this study based on the pull-out test. The obtained nonlinear governing equations were dimensionless, which are expressed in finite difference form. The accuracy of the numerical solution was tested by varying the number of elements, and the results indicated that discretizing the reinforcement length into 300 units provides an accurate numerical solution within a reasonable computational time.
2. Considering the trilinear shear stress displacement form of geotextile reinforced tailings, three new dimensionless interaction terms, namely, the relative stiffness *α*, relative displacement *β*, and relative interface shear stiffness *η* of the reinforcement-tailings interface, were introduced. A method for predicting the approximate value of *α* in the low-tensile-force displacement range was proposed, and the normalized tensile force and displacement responses were obtained for different *α* and *β* ranges.
3. The predicted pull-out displacements were compared with the pull-out test results, and it was concluded that the proposed model can accurately predict the pull-out properties of extensible geotextile-reinforced tailings. Moreover, the interaction parameters of *k*_s1_, *α*, and *β* were accurately estimated to further improve the predictability of the test results.
